# Supplementary figures and images for: Identification of key genes and pathways associated with cholangiocarcinoma development based on weighted gene correlation network analysis
Source: PeerJ. 2019 Oct 31;7:e7968. doi: 10.7717/peerj.7968 (PMC6825751; doi:10.7717/peerj.7968)

### Scale independence

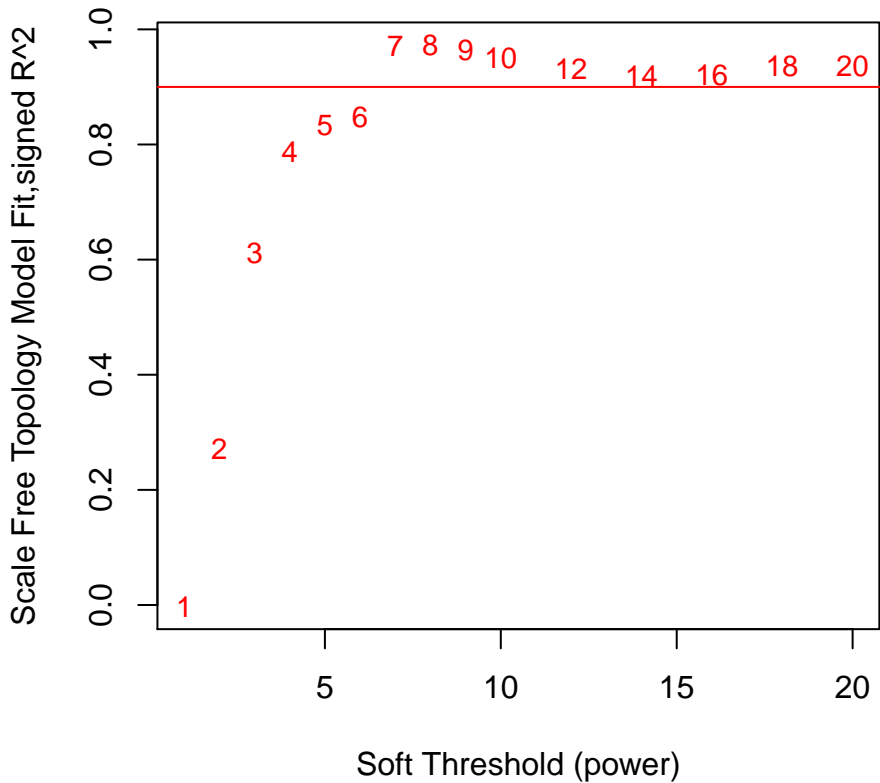

### Mean connectivity

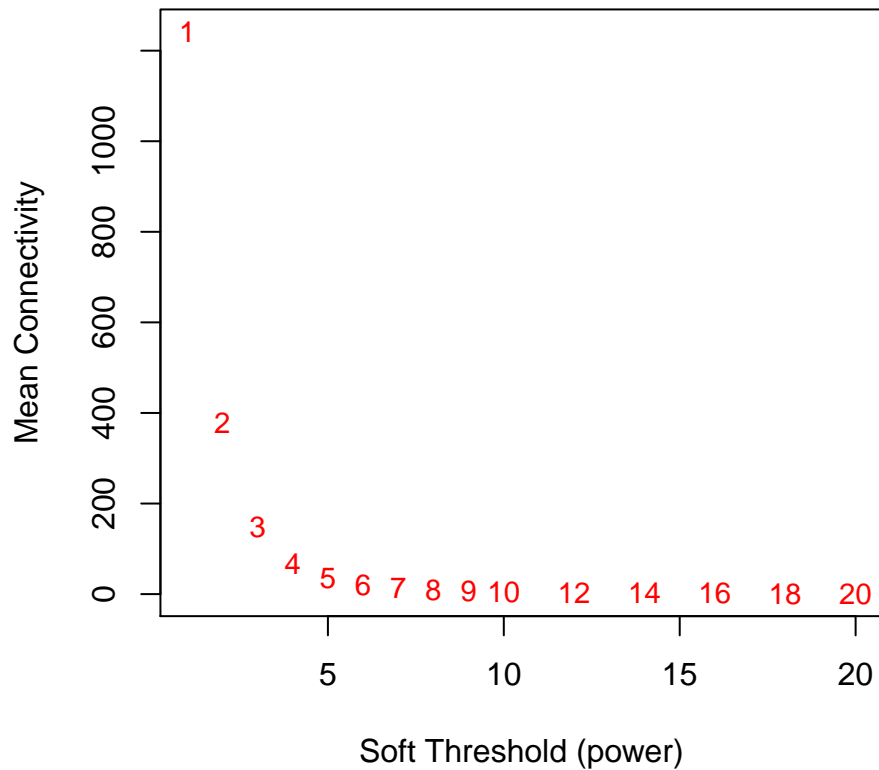

Supplement: Supplemental Information 1 [file peerj-07-7968-s001.pdf]
